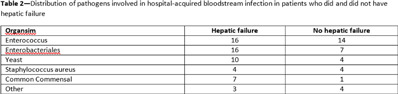# Hospital-acquired bloodstream infections in patients with and without hepatic failure

**DOI:** 10.1017/ash.2022.115

**Published:** 2022-05-16

**Authors:** Jordan Bosco, Patrick Burke, Francisco Marco Canosa, Stephen Wilson, Steven Gordon, Thomas Fraser

## Abstract

**Background:** The NHSN parameter estimate for predicted number of central-line–associated bloodstream infection (CLABSI) is the same for gastroenterology wards as other specialty wards, such as behavioral health and gerontology. We conducted this study to contribute to the body of knowledge surrounding the risk for hospital-acquired bloodstream infection (HABSI) in patients with and without hepatic failure. The Cleveland Clinic is a 1,200-bed, multispecialty hospital with a solid-organ transplant service. Patients with hepatic failure who do not require critical care are housed on 36-bed unit A. On unit A, 43% of patients are under hepatology or gastroenterology service, although 51% of patients are under general internal medicine. Overall, unit A has a high incidence of HABSI. **Methods:** Surveillance for HABSI and CLABSI is performed at the Cleveland Clinic per NHSN protocol. All patients with a midnight stay on unit A from January 2019 through September 2021 were dichotomized as having hepatic failure (yes or no) if they ever received the *International Classification of Diseases Tenth Revision* code for “hepatic failure, not elsewhere classified.” We joined the diagnostic code to patient days and central-line-days databases and summarized the data using Microsoft Excel software. We stratified the number of patients, patient days, device days, infection classification, and hospital length of stay by whether the patient had hepatic failure, and we compared the incidence of HABSI and CLABSI between the 2 groups using OpenEpi version 3.01 software. **Results:** We identified 72 HABSIs among 4,285 patients who stayed on unit A for 30,910 patient days during the study period. The incidences of HABSI in patients with and without hepatic failure were 39.0 and 13.9 per 10,000 patient days, respectively (*P* < .001). The incidence of CLABSI was 5.4 and 1.9 per 1,000 line days, respectively (*P* = .01). Patients with hepatic failure stayed longer (11.5 vs 5.9 days), yet the central-line utilization ratios were not substantially different (0.25 vs 0.24). *Enterococcus* was the most common pathogen involved in CLABSI in both groups (Table 2). **Conclusions:** Patients with hepatic failure experienced CLABSI more frequently than patients without hepatic failure, stayed longer in the hospital, and were less likely have HABSI attributed to another primary focus of infection according to NHSN definitions. Although hepatic failure may be among the most severe conditions among patients in a gastroenterology ward, we have demonstrated that these units house a population uniquely susceptible to HABSI and CLABSI.

**Funding:** None

**Disclosures:** None

**Table:**
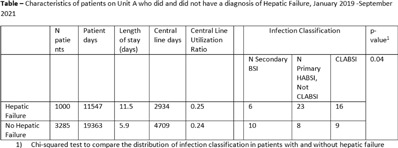

**Table:**